# Helmsman: fast and efficient mutation signature analysis for massive sequencing datasets

**DOI:** 10.1186/s12864-018-5264-y

**Published:** 2018-11-28

**Authors:** Jedidiah Carlson, Jun Z. Li, Sebastian Zöllner

**Affiliations:** 1 Department of Computational Medicine & Bioinformatics, University of Michigan, Ann Arbor, MI USA; 2 Department of Human Genetics, University of Michigan, Ann Arbor, MI USA; 3 Department of Biostatistics, University of Michigan, Ann Arbor, MI USA; 4 Department of Psychiatry, University of Michigan, Ann Arbor, MI USA; 5 Department of Genome Sciences, University of Washington, Seattle, WA USA

**Keywords:** Mutational signatures, Cancer genomics, Python, Single nucleotide variants

## Abstract

**Background:**

The spectrum of somatic single-nucleotide variants in cancer genomes often reflects the signatures of multiple distinct mutational processes, which can provide clinically actionable insights into cancer etiology. Existing software tools for identifying and evaluating these mutational signatures do not scale to analyze large datasets containing thousands of individuals or millions of variants.

**Results:**

We introduce Helmsman, a program designed to perform mutation signature analysis on arbitrarily large sequencing datasets. Helmsman is up to 300 times faster than existing software. Helmsman’s memory usage is independent of the number of variants, resulting in a small enough memory footprint to analyze datasets that would otherwise exceed the memory limitations of other programs.

**Conclusions:**

Helmsman is a computationally efficient tool that enables users to evaluate mutational signatures in massive sequencing datasets that are otherwise intractable with existing software. Helmsman is freely available at https://github.com/carjed/helmsman.

**Electronic supplementary material:**

The online version of this article (10.1186/s12864-018-5264-y) contains supplementary material, which is available to authorized users.

## Background

The spectrum of somatic single-nucleotide variants (SNVs) in cancer genomes carries important information about the underlying mutation mechanisms, providing insight into the development, evolution, and etiology of the cancer cell populations [1]. Evaluating these patterns of variation, referred to as “mutational signatures,” has become an important task in precision oncology, as mutational signatures can be used both to refine cancer diagnoses and identify effective targeted therapies [2].

Several software programs and web services have been developed to identify and evaluate the mutational signatures present in cancer genomes [3–7]. Most methods consider 96 mutation subtypes, defined by the type of base change (C>A, C>G, C>T, T>A, T>C, T>G) and the trinucleotide sequence context (e.g., C[T>G]T, C[C>A]T, and so on) [8]. Mutation signature analysis methods express the observed mutation spectrum in each sample as a linear combination of a preset number (K) of distinct mutational signatures, where the signatures are inferred directly from the input data, or taken from external sources such as the COSMIC mutational signature database [1]. These programs typically start with an input file, often in a standard format such as Variant Call Format (VCF) or Mutation Annotation Format (MAF), containing the genomic coordinates of each SNV and the sample(s) in which they occur. As a first step, these SNVs must be summarized into a NxS mutation spectra matrix, M, containing the frequencies of S different SNV subtypes in each of N unique samples (where the *M*_*i*, *j*_ entry indicates the number of observed SNVs of subtype j in sample i). Most methods are implemented as R packages and must read the entire input file into memory prior to generating the mutation spectra matrix. For large input files, containing for example millions of SNVs and hundreds or thousands of samples, the memory required for this step can easily exceed the physical memory capacity of most servers, rendering such tools incapable of directly analyzing large datasets. To circumvent these computational bottlenecks, researchers must either limit their analyses to small samples, pool samples together, or develop new software to generate the mutation spectra matrix. Presently, the largest studies to perform mutation signature analysis have included millions of mutations in thousands of whole cancer genomes [1, 9], but these studies have pooled individual samples into ~ 30 distinct cancer types, potentially obscuring the presence of mutation signatures unique to individual cancer genomes or more granularly defined cancer types.

## Implementation

To overcome the limitations of existing mutation signature analysis tools, we have developed Helmsman, a new mutation signature analysis program optimized for performing mutation signature analysis on arbitrarily large datasets. Helmsman is implemented in Python, and is primarily designed to accept VCF files as input (though Helmsman can also accept data in other formats, such as MAF). Helmsman uses the powerful cyvcf2 Python library [10] for back-end processing of VCF files. Cyvcf2 is essentially a Python wrapper for the same htslib C libraries that serve as the back-end for standalone compiled VCF-processing toolkits like bcftools [11], and offers comparable speed and memory efficiency [10].

### Generation of mutation spectra matrices

For each SNV in a VCF file, Helmsman defines the mutation type based on the reference and alternative alleles, then queries the corresponding reference genome for the trinucleotide context of the SNV, determining subtype *j*. The functions for querying the reference genome were derived from the pyfaidx Python library, which provides fast and memory-efficient random access to reference genome files, without requiring the entire file to be loaded into memory [12]. The genotypes of the *N* samples for this SNV are represented as an integer array, with the number of alternative alleles per sample coded as 0, 1, or 2 according to the observed genotype [10]. Based on the genotype for sample *i*, Helmsman increments the *M*_*i*, *j*_ entry of the mutation spectra matrix accordingly (i.e., if individual *i* is heterozygous, *M*_*i*, *j*_ is incremented by 1, but if individual *i* is homozygous for the reference allele, *M*_*i*, *j*_ remains unchanged). This procedure is fully vectorized, meaning that instead of performing N sequential operations (i.e., looping through the genotypes of the N samples for a given SNV and adding the value of each element to the corresponding *M*_*i*, *j*_ entry), Helmsman performs element-wise addition of the genotype array to the jth column of the M matrix in a single computational operation. Consequently, Helmsman’s processing time is independent of sample size and scales linearly with the number of SNVs. The only objects stored in memory at any given moment are the array of N genotypes for the SNV being processed and the Nx96 mutation spectra matrix, so memory usage is independent of the number of SNVs and scales linearly with sample size.

### Mutation signature analysis

Once the mutation spectra matrix has been generated, Helmsman can apply non-negative matrix factorization (NMF) to this matrix to infer the underlying mutation signatures and their loadings within each sample, using functions from the nimfa [13] Python library. Alternatively, Helmsman can perform principal component analysis (PCA) to the mutation spectra matrix using functions from the scikit-learn [14] library. We note that because PCA does not enforce non-negativity, the resulting components do not have a useful biological interpretation like the NMF signatures do [15]; however, PCA remains useful as an orthogonal exploratory analysis to highlight patterns of similarity in the spectra of the samples (as in [16]), which can help guide understanding of how many distinct signatures may be contributing to the observed mutation spectra in a given dataset [17].

Alternatively, users can forgo the built-in mutation signature analysis and instead opt to write the mutation spectra matrix to a file and perform downstream analyses in their preferred environment. When this option is selected, users can specify one of several different R packages that they wish to use for their downstream analysis (e.g., SomaticSignatures or deconstructSigs) and Helmsman will automatically generate an R script with all code necessary to load the output matrix into R and format it for compatibility with the specified package. This feature was designed expressly to enable and encourage researchers to continue using existing mutation signature analysis tools that would otherwise be incapable of processing large datasets due to computational bottlenecks. Further, this feature better enables users to perform multiple complementary analyses. For example, after generating the mutation spectra matrix with Helmsman, users could perform supervised signature decomposition with deconstructSigs [4] to assess the presence of known signatures, then apply the de novo signature extraction methods of SomaticSignatures [3] or signeR [5] to determine if the data contain novel signatures, without ever needing to re-generate or manually reformat the mutation spectra matrix.

### Additional features

In addition to being optimized for speed and low memory usage, Helmsman includes several features to accommodate various usage scenarios and minimize the amount of pre-processing necessary to analyze large mutation datasets. For example, if input data are spread across multiple files (e.g., by different sub-samples or genomic regions), Helmsman can process these files in parallel and aggregate them into a single mutation spectra matrix, providing additional performance improvements and avoiding the need to generate intermediate files. Similarly, in certain applications, it may be desirable to pool similar samples together (e.g., by tumor type) when generating the mutation spectra matrix. Helmsman can pool samples on-the-fly, without needing to pre-annotate or reshape the input file with the desired grouping variable. All features are described in detail in the online documentation.

### Alternative deployment options

We have also created a Docker image to easily deploy Helmsman in an isolated and reproducible environment on virtually any personal computer, local server, or cloud server where Docker containers are supported. This Docker image contains the Helmsman source code along with a minimal Python environment and all the necessary library dependencies, as well as the bcftools suite [11] to perform any necessary pre-processing within the same container environment. When the Docker container is deployed, an interactive Jupyter notebook will be available to run Helmsman. Further, this Docker image is fully compatible with the Binder platform [18], which effectively enables Helmsman to be used as a web server for performing mutation signature analysis on small datasets. A Binder instance of Helmsman can be accessed at https://mybinder.org/v2/gh/carjed/helmsman/master.

## Results

### Performance comparison

We compared Helmsman’s performance to that of three published R packages: SomaticSignatures [3], deconstructSigs [4], and signeR [5]. We also considered several other tools, and discuss their performance in the Additional file 1. For our tests, we generated a small VCF file (2.7 MB compressed with bgzip) containing 15,971 germline SNVs on chromosome 22 from 2504 samples sequenced in the 1000 Genomes Project phase 3 [19], and measured the runtime and memory usage necessary for each program to generate the mutation spectra matrix. We also attempted to run each program using the full chromosome 22 VCF file from the 1000 Genomes Project, containing 1,055,454 SNVs in 2504 individuals. The number of SNVs in this VCF file is comparable to those of the large somatic SNV datasets analyzed in [1, 9].

**Fig. 1:**
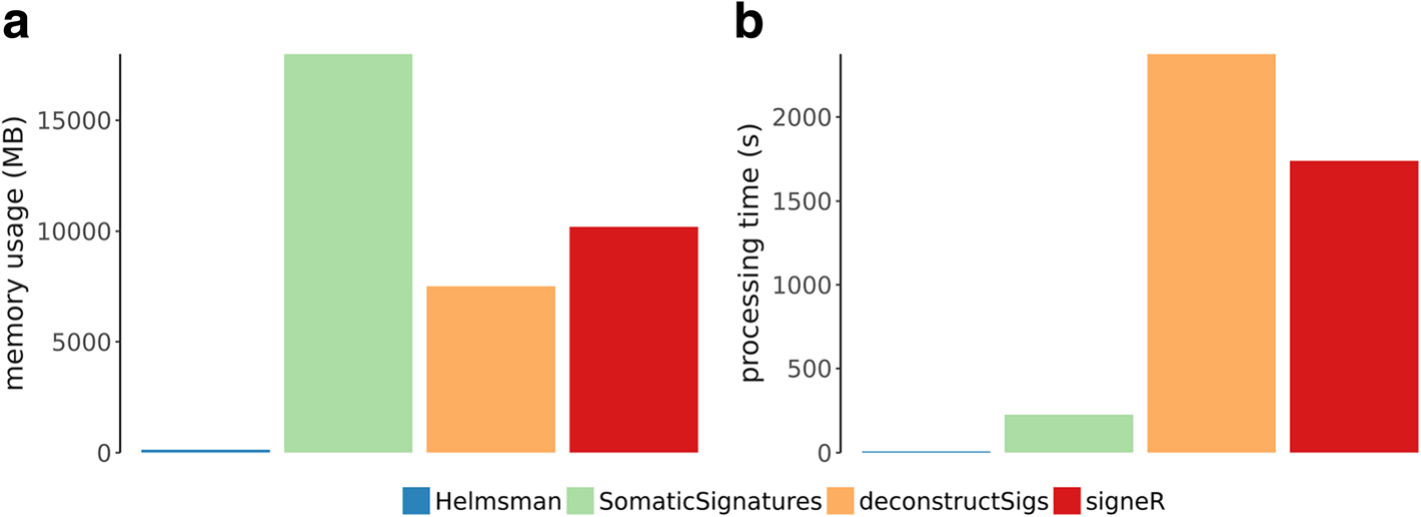
Performance comparison for generation of the mutation spectra matrix by different programs. For Helmsman and three other mutation signature analysis tools (SomaticSignatures, deconstructSigs, and signeR), we measured the maximum memory usage in megabytes (**a**) and processing time in seconds (**b**) required to generate the 2504 × 96 mutation spectra matrix from a VCF file containing 15,971 SNVs in 2504 samples from the 1000 Genomes project

All programs generated the same mutation spectra matrices. Helmsman processed the small VCF file in 8 s, with a memory footprint of 140 MB, and the full VCF file in 482 s (corresponding to a linear increase for ~60x more variants) with no increase in memory usage as the sample size remained the same. In contrast, to process the small VCF file, SomaticSignatures took 227 s with a memory footprint of 18GB, deconstructSigs took 2376 s and 7.5GB of memory, and signeR took 1740 s and 10.2GB of memory (Fig. 1). None of these R packages were able to load the full VCF file due to memory allocation errors. All other tools we considered showed similar performance bottlenecks when compared to Helmsman (Additional file 1, Additional file 2: Figure S1).

To further highlight the speed and efficiency of Helmsman for large datasets, we evaluated the entire set of 36,820,990 autosomal biallelic SNVs from the 1000 Genomes phase 3 dataset (14.4 GB when compressed with bgzip). Using 22 CPUs (one per chromosome VCF file), Helmsman generated the mutation spectra matrix in 64 min (approximately 1.5 s per sample), with each process requiring < 200 MB of memory.

### Validating mutation signatures

Because Helmsman uses NMF algorithms from the nimfa Python library [13] rather than the NMF R package [20] (as used in the SomaticSignatures [3] and MutSpec [21] programs), we also evaluated whether the mutational signatures inferred by our implementation were consistent with biologically validated signatures described in the literature. To this end, we analyzed a dataset of 100 simulated mutation spectra, which were randomly generated by Rosenthal et al. [4] using linear combinations of the 27 mutation signatures inferred from 7042 cancer samples published by Alexandrov et al. [1]. We reasoned that if our implementation of the NMF signature decomposition method is valid, the signatures inferred by Helmsman should reflect the underlying signatures from [1] by which these data were simulated. We found that the mutational signatures inferred by Helmsman corresponded closely to these known signatures (Additional file 3: Figure S2), demonstrating that Helmsman’s implementation of the NMF algorithm performs as expected, and is comparable to other NMF-based de novo signature extraction methods.

## Conclusions

As massive sequencing datasets become increasingly common in areas of cancer genomics and precision oncology, there is a growing need for software tools that scale accordingly and can be integrated into automated workflows. Our program, Helmsman, provides an efficient, standardized framework for performing mutation signature analysis on arbitrarily large, multi-sample VCF or MAF files. For small datasets, Helmsman performs this task up to 300 times faster than existing methods, and is the only tool that can be directly applied to modern large sequencing datasets.

## Availability and requirements

Project name: Helmsman

Project home page: https://github.com/carjed/helmsman

Operating system: Platform independent

Programming language: Python

Other requirements: None

License: MIT License

Any restrictions to use by non-academics: no restriction

## Additional files

Additional file 1: Assessment of the performance of other mutation signature analysis tools. (PDF 87 kb)

Additional file 2: **Figure S1.** Performance comparison for generation of the mutation spectra matrix from a MAF input file. (PDF 89 kb)

Additional file 3: **Figure S2.** Evaluation of mutation signatures inferred by Helmsman. (PDF 250 kb)

## Abbreviations

MAF: Mutation annotation format
NMF: Non-negative matrix factorization
PCA: Principal component analysis
SNV: Single nucleotide variant
VCF: Variant call format
