# Supplementary material for: Helmsman: fast and efficient mutation signature analysis for massive sequencing datasets
Source: BMC Genomics. 2018 Nov 28;19:845. doi: 10.1186/s12864-018-5264-y (PMC6263557; doi:10.1186/s12864-018-5264-y)
Supplement: Supplementary file 1 — Assessment of the performance of other mutation signature analysis tools. (PDF 87 kb) [file 12864_2018_5264_MOESM1_ESM.pdf]

Supplementary Material for Helmsman: fast and efficient mutation signature analysis for massive sequencing datasets

Performance of other mutation signature analysis tools

In addition to the R packages we evaluated in the main paper, we considered several other mutation signature analysis tools which provide functions for generating mutation spectra matrices from VCF or Mutation Annotation Format (MAF) files. Where applicable, we used the small and full 1000 Genomes chromosome 22 VCF files described in the main paper. In our tests, each of these tools showed substantial performance bottlenecks compared to Helmsman, or were subject to other limitations that made them infeasible for applying to our test datasets.

Mutagene and Mutalisk

Mutagene [1] and Mutalisk [2] are implemented as web servers and provide graphical interfaces for users to upload their data, with all data processing performed on the server end. We successfully uploaded the 158.6MB uncompressed small VCF file to Mutalisk, and the uploading and processing took approximately 60 seconds (compared to 8 seconds with Helmsman). Mutagene would not accept the small VCF file in either compressed or uncompressed format. Neither tool would accept the full VCF file when we attempted to upload it. Moreover, although Mutalisk at first appeared to offer reasonably fast performance for the small VCF file, we found that it did not properly parse the data into 2,504 unique samples as expected, and incorrectly assumed the SNVs were all from a single sample. Mutalisk does allow

users to upload multiple single-sample VCF files, but limits input to 300 files, and is therefore only feasible for relatively small sample sizes.

MutSpec

MutSpec is implemented as a Galaxy toolbox, enabling users with limited programming expertise to perform mutation signature analysis with a graphical interface [3]. Though we did not have a Galaxy server available to directly evaluate MutSpec's performance on our test datasets, the authors reported that it takes ~7 minutes to annotate a VCF file containing 100,000 variants (in an unstated sample size) using 24 CPUs, and 4 hours using a single CPU. Assuming MutSpec's runtime scales linearly with the number of SNVs, we estimate that it would take at least 60 seconds using 24 CPUs and over 40 minutes using a single CPU to parse the 15,971 SNVs in the small VCF file, compared to 8 seconds on a single CPU when using Helmsman. Similarly, to parse the 1,055,454 SNVs contained in the full chromosome 22 VCF file used in our tests, we estimate that MutSpec would take over an hour when using 24 CPUs, and over 40 hours on a single CPU, compared to 8 minutes on a single CPU when using Helmsman.

We note that these performance estimates for MutSpec are based on the reported runtime only for the annotation step of the MutSpec pipeline, which generates an intermediate tab-delimited file containing functional and structural annotations for each SNV in the input VCF file. Our estimates did not take into account the additional processing time required to parse this intermediate file into the Nx96 mutation spectra matrix, so our estimates represent a lower bound for the runtime necessary to generate the mutation spectra matrix using MutSpec.

Maftools and Mutation-Signatures

Somatic mutation data are sometimes represented in Mutation Annotation Format (MAF) files, a tab-delimited format with one variant per row, and several dozen additional annotation columns (described in detail at https://docs.gdc.cancer.gov/Data/File_Formats/MAF_Format/). Unlike VCF files, MAF files do not indicate the genotypes of each individual in the sample, so a variant present in two or more individuals must be indicated as multiple rows. We considered two programs designed specifically for applying mutation signature analysis to MAF files: maftools, an R package [4], and Mutation-Signatures, an unpublished collection of Python scripts developed by researchers at Memorial Sloan Kettering Cancer Center in New York, NY (https://github.com/mskcc/mutation-signatures).

We evaluated the performance of these MAF-specific tools using a MAF file with data from 377 Liver Hepatocellular Carcinoma (LIHC) samples, available from The Cancer Genome Atlas at https://portal.gdc.cancer.gov/legacy-archive/files/15ce66c6-0211-4f03-bd41-568d0818a044. This file was 1.4GB in size, and contained 60,691 somatic SNVs (interspersed with 1,415,224 non-SNV variants that are not considered in this type of analysis).

Helmsman generated the mutation spectra matrix from this MAF file in 96 seconds and required less than 130MB of memory (**Additional File 2: Fig. S1**). Both Mutation-Signatures and maftools generated output identical to that of Helmsman. Mutation-Signatures performs this task in two steps, first creating an intermediate MAF file with each SNV annotated with the surrounding trinucleotide context, then parsing this file to generate the mutation spectra matrix

and, in the same step, performing supervised decomposition of each sample into 30 pre-specified signatures. Mutation-Signatures took a total of 402 seconds to run these scripts (145 seconds to generate the intermediate MAF file, and 377 seconds to generate the mutation spectra matrix and perform the signature decomposition), with a maximum memory footprint of 6.5GB (memory usage peaked when generating the intermediate MAF file) (**Additional File 2: Fig. S1**). Maftools took 207 seconds and required 9.2GB of memory to read the same input MAF file and generate the mutation spectra matrix, using the functions `read.maf` and `trinucleotideMatrix`, respectively (**Additional File 2: Fig. S1**). Like the VCF-specific R packages we evaluated in the main paper, we note that maftools is memory-intensive, even for relatively small input files, and susceptible to memory bottlenecks as the input file size increases. Maftools' high memory usage and longer processing time is largely attributable to the inherently high dimensionality of MAF files: although only five columns (Chromosome, Position, Reference Allele, Alternative Allele, and Sample ID) are necessary to generate the mutation spectra matrix, Maftools requires the input MAF file to contain many additional mandatory columns.

References

1. Goncearenco A, Rager SL, Li M, Sang Q-X, Rogozin IB, Panchenko AR. Exploring background mutational processes to decipher cancer genetic heterogeneity. Nucleic Acids Res. 2017;45:W514–22.

2. Lee J, Lee AJ, Lee J-K, Park J, Kwon Y, Park S, et al. Mutalisk: a web-based somatic MUTation AnaLyIS toolKit for genomic, transcriptional and epigenomic signatures. Nucleic Acids Res. 2018. doi:10.1093/nar/gky406.

3. Ardin M, Cahais V, Castells X, Bouaoun L, Byrnes G, Herceg Z, et al. MutSpec: a Galaxy toolbox for streamlined analyses of somatic mutation spectra in human and mouse cancer genomes. BMC Bioinformatics. 2016;17:170.

4. Mayakonda A, Phillip Koeffler H. Maftools: Efficient analysis, visualization and summarization of MAF files from large-scale cohort based cancer studies. bioRxiv. 2016;:052662. doi:10.1101/052662.
