# Supplementary material for: Helmsman: fast and efficient mutation signature analysis for massive sequencing datasets
Source: BMC Genomics. 2018 Nov 28;19:845. doi: 10.1186/s12864-018-5264-y (PMC6263557; doi:10.1186/s12864-018-5264-y)
Supplement: Supplementary file 2 — Figure S1. Performance comparison for generation of the mutation spectra matrix from a MAF input file. (PDF 89 kb) [file 12864_2018_5264_MOESM2_ESM.pdf]

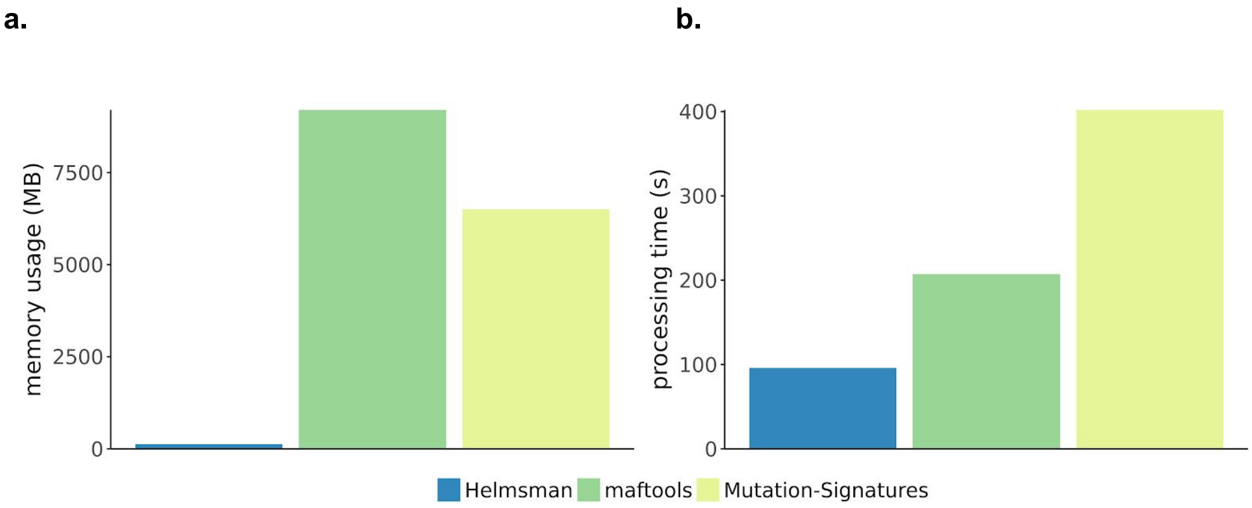

Figure S1. Performance comparison for generation of the mutation spectra matrix from a MAF input file. For Helmsman and two other MAF-specific mutation signature analysis tools (maftools and Mutation-Signatures), we measured the maximum memory usage in megabytes (**a**) and processing time in seconds (**b**) required to generate the 377 x 96 mutation spectra matrix from a MAF file containing 60,691 SNVs (in addition to 1,415,224 non-SNV variants that were present in the file but not analyzed) in 377 samples from The Cancer Genome Atlas.
