# Supplementary material for: Helmsman: fast and efficient mutation signature analysis for massive sequencing datasets
Source: BMC Genomics. 2018 Nov 28;19:845. doi: 10.1186/s12864-018-5264-y (PMC6263557; doi:10.1186/s12864-018-5264-y)
Supplement: Supplementary file 3 — Figure S2. Evaluation of mutation signatures inferred by Helmsman. (PDF 250 kb) [file 12864_2018_5264_MOESM3_ESM.pdf]

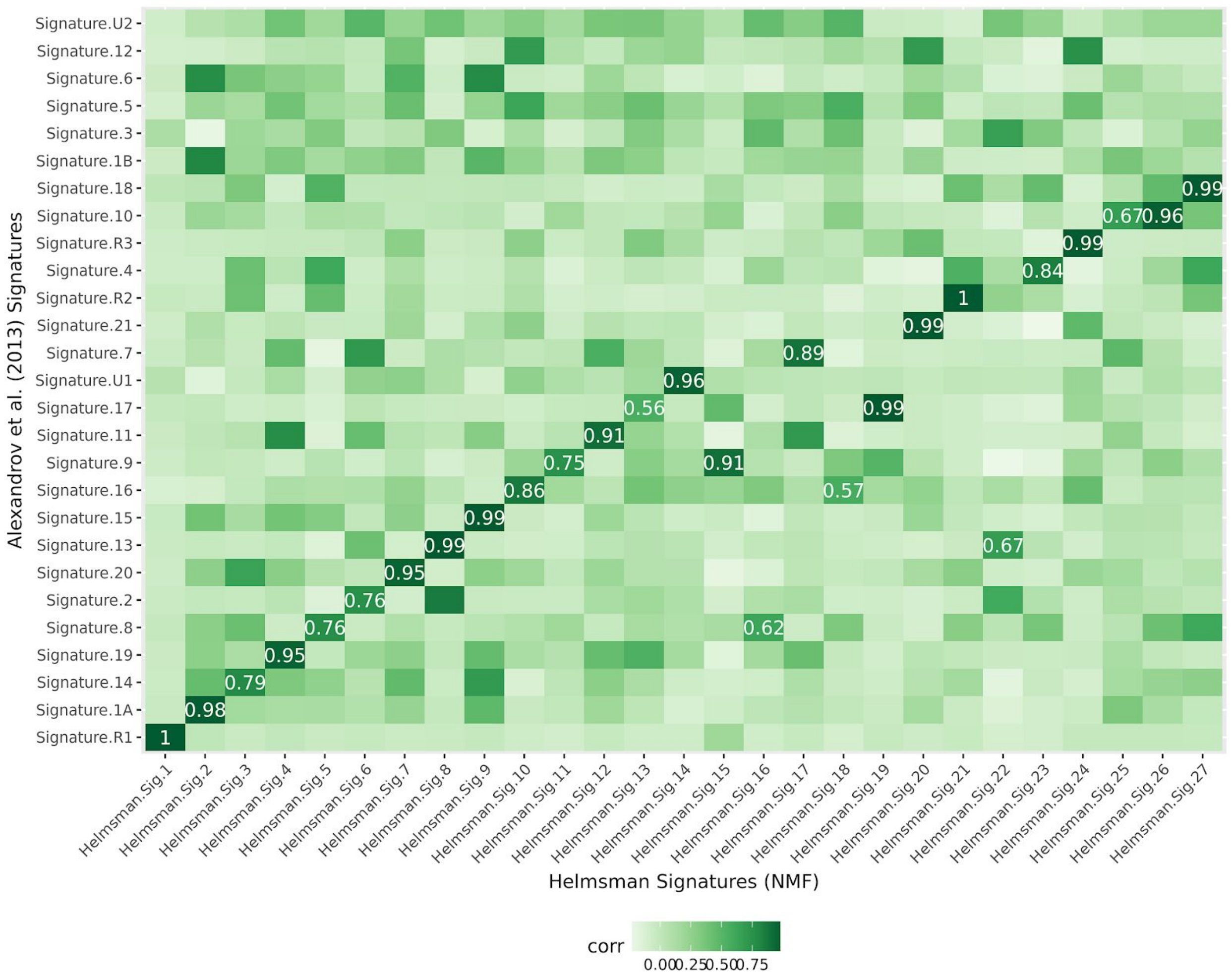

Figure S2. Evaluation of mutation signatures inferred by Helmsman. Using simulated mutation spectra for 100 samples, generated by Rosenthal et al. [1] as random linear combinations of the 27 mutational signatures described by Alexandrov et al. [2], we applied Helmsman with a rank 27 NMF decomposition to perform de novo signature extraction. This heatmap shows the Pearson correlation between each of these 27 inferred signatures (x axis) with the 27 known signatures on which these samples were simulated (y axis). For each of the Helmsman signatures, the Alexandrov signature with the strongest correlation is indicated in the cell containing text. 21 of the 27 Helmsman signatures appear to be very strongly correlated with just a single Alexandrov signature (r>0.75), demonstrating that Helmsman's de novo signature extraction recovers the expected mutational signatures.

References

1. Rosenthal R, McGranahan N, Herrero J, Taylor BS, Swanton C. DeconstructSigs: delineating mutational processes in single tumors distinguishes DNA repair deficiencies and patterns of carcinoma evolution. Genome Biol. 2016;17:31.

2. Alexandrov LB, Nik-Zainal S, Wedge DC, Aparicio SAJR, Behjati S, Biankin AV, et al. Signatures of mutational processes in human cancer. Nature. 2013;500:415–21.
